# A Pilot Feasibility Study to Establish Full Pulpotomy in Mature Permanent Teeth with Symptomatic Irreversible Pulpitis as a Routine Treatment in Mexican Public Healthcare Services

**DOI:** 10.3390/healthcare10122350

**Published:** 2022-11-23

**Authors:** Roberto Gustavo Sánchez-Lara y Tajonar, Jaime Vicente Vergara-Tinoco, Till Dammaschke, Rubén Abraham Domínguez-Pérez

**Affiliations:** 1 Department of Endodontics, Faculty of Medicine, Universidad Autónoma de Querétaro, Santiago de Querétaro 76176, Mexico; 2 Social Service Intern of the Public Dentalcare Clinic “La Negreta”, Jurisdicción Sanitaria N° 1, del Estado de Querétaro 76070, Mexico; 3 Department of Periodontology and Operative Dentistry, Westphalian Wilhelms-University, 48149 Münster, Germany; 4 Laboratory of Multidisciplinary Dentistry Research, Faculty of Medicine, Universidad Autónoma de Querétaro, Santiago de Querétaro 76176, Mexico

**Keywords:** full pulpotomy, irreversible pulpitis, permanent teeth, public health system, routine treatment

## Abstract

Symptomatic irreversible pulpitis is a common dental disease for which root canal treatment (RCT) has been the standard treatment. However, in many countries, RCT is considered a high-cost treatment that is not covered by public healthcare services; this forces patients to have dental extraction as their only option to relieve pain. In the last decade, several investigations have provided evidence that an alternative treatment known as full pulpotomy (FP) could be an alternative for patients who could not afford the cost of an RCT. Nevertheless, evidence is lacking on the success rate that could be obtained if it is performed in a public dental care clinic (PDCC). The present investigation has two main objectives. To be the first approach of a multicentric feasibility study to find out whether an FP performed by a general practice dentist (GPD) in a PDCC could be suitable and establish its success rate and patient satisfaction. Patients attending a PDCC with symptoms of irreversible pulpitis were invited to participate. FP was performed and followed up at 1, 3, 6, 9, and 12 months. The treatment success was assessed by combining three variables, patient satisfaction, clinical, and radiographic outcomes. Forty-one patients from 17 to 78 years old received the intervention. In total, 97.5% were completely satisfied with the treatment and were considered successful since none of the clinical or radiographic variables were present in any of the follow-ups. An FP performed by a GPD in a PDCC could be suitable as a routine treatment for symptomatic irreversible pulpitis due to the excellent success rate and patient satisfaction.

## 1. Introduction

In Mexico, a country with more than 126 million inhabitants, 93.3% of adults suffer from dental caries [1]. Untreated caries and extensive restorative procedures will invariably result in irreversible pulpitis. Mexican public healthcare services, as in many other countries, focus mainly on prevention, with programs that seek to promote oral care and primary attention programs with a limited number of treatments that can be covered. In patients with irreversible pulpitis, the need for a root canal treatment (RCT) can only be indicated but not provided as it is a technically demanding procedure that causes high economic costs. The high prices result from the need for sophisticated equipment and specialized clinicians, whereby the treatment cannot be afforded and provided by public healthcare services. In addition, root canal-treated teeth usually require a more complex final restoration than resin composite or amalgam fillings (the alternative that Mexican public healthcare services can offer). Therefore, the treatment can only be provided as a private service that has to be fully paid for by the patient. In Mexico, an RCT with its respective final restoration costs MXN 4000 ± 1000 pesos (USD 200 ± 50), while the monthly income of 36% of the population is at a maximum of MXN 3780 pesos (USD 189) [2]. This definitely makes it impossible for at least one-third of the population to undergo an RCT and indirectly forces them to decide on tooth extraction to relieve pain and discomfort [3]. Public dental care clinics (PDCC) offer no-cost extractions, whereas in private practice, the extractions cost around MXN 500 pesos (USD 25). 

It is well known that financial constraints affect oral and general health. Tooth extraction is a typical result of irreversible pulpitis [4]; evidently, it does not represent an optimal treatment option because it can considerably influence chewing ability, appearance, phonics, nutrition, and consequently, the quality of life [5,6]. In the last decade, vital pulp therapies have become more relevant in cases of irreversible pulpitis as the treatments are significantly less invasive and more affordable. These conservative pulp management therapies have regained favor since developing and using hydraulic calcium silicate-based cement such as mineral trioxide aggregate (MTA). MTA is a widely used biomaterial for vital pulp therapies [7]; it is the gold standard for pulp capping due to its predictable pulp tissue regeneration [8]. Several reports have demonstrated its successful use for pulpotomies in primary [9,10,11,12] or immature permanent teeth [13,14,15] and permanent mature teeth (closed apices) with irreversible pulpitis or carious exposure [16,17,18,19,20,21,22,23]. 

In addition to the lower economic costs of such vital pulp therapies, including full pulpotomy (FP), other advantages come with them such as the reduction in operative time, the preservation of tooth structure [24], as well as the proprioceptive mechanisms that could reduce the propensity of a tooth fracture [25,26], mainly because they only involve the removal of the coronal pulp to the level of canal orifices and keep radicular pulp vital. FP has long been considered a definitive treatment for deciduous teeth with pulp inflammation [27,28] and permanent immature teeth diagnosed with reversible or irreversible pulpitis [20,29]. However, until recently, it has not been considered a routine option for irreversible pulpitis in mature permanent teeth. Several investigations have provided evidence that this conservative treatment could be an alternative to RCT or tooth extraction [20,22,30,31,32,33] for patients who could not afford the cost of RCT. However, significant differences exist in the published protocols, for example, the operator’s background (endodontist, postgraduate students, general practitioners), the place where the treatment is performed (hospital, university, private practice), and the available clinical armamentarium. 

It is an interesting consideration to offer FP as a routine treatment for symptomatic irreversible pulpitis or carious pulp exposure in the PDCC of Mexican public healthcare services to the population that cannot afford an RCT. However, to formally raise this proposal, it is necessary to evidence the possibility of it being undertaken repeatedly by general practice dentists (GPD) using the available instruments, materials, and a workflow with an adequate success rate and patient satisfaction through the realization of a multicentric feasibility study, but first, a single center feasibility study (pilot study) is necessary. Then, the present investigation has two main objectives. It is the first approach of a multicentric feasibility study to find out whether the FP performed by a GPD in a PDCC of the Mexican public healthcare services could be suitable and establish the success rate of this proposal and patient satisfaction. 

## 2. Materials and Methods

This investigation was designed as a longitudinal prospective single-arm cohort study. The sample size was established by convenience and based on similar sample sizes in studies investigating the outcome of vital pulp therapies after at least one year. Ethical approval (code number: JS1/ENS/450/19) was obtained from the bioethics committee of the Ministry of Health of the State of Querétaro (Mexico), and the authorization was granted to perform the procedures in the “Centro de Salud -La Negreta- Jurisdicción Sanitaria N° 1 del Estado de Querétaro”. 

### 2.1. Training to the General Practice Dentist (GPD)

One GPD (J.V.V.T.) was theoretically and practically trained by an experienced endodontist (R.G.S.L.T.) in a 5 h lesson. Five fundamental topics were covered. 1. Pulp diagnosis, including pulp sensitivity tests, 2. Access cavity preparation with emphasis on the complete removal of the pulp chamber roof, 3. Hemostasis and application of the MTA with emphasis on adequate thickness and compaction, 4. Final restoration with amalgam, and 5. Explanations to the patient to solve doubts.

### 2.2. Patient Selection

**Figure 1:**
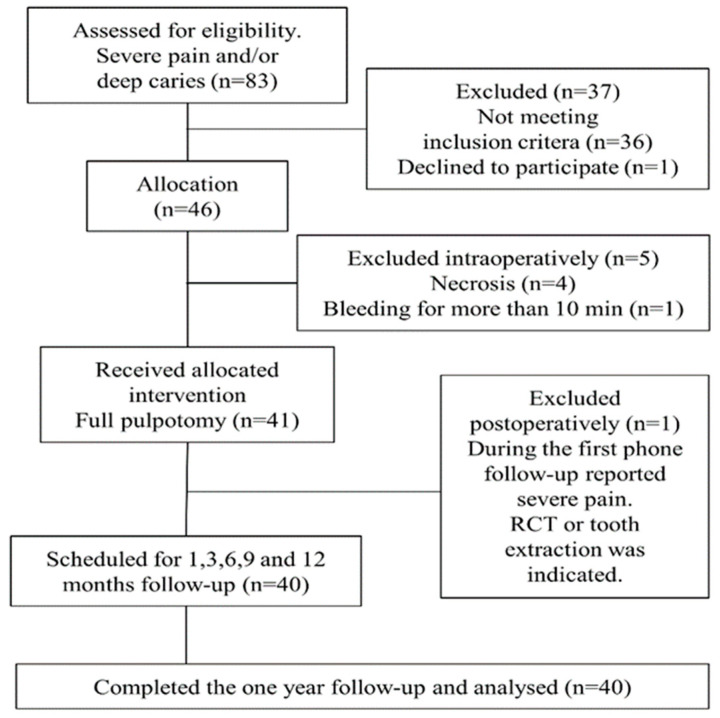
Flow diagram of the eligible patients up to 12-month follow-up.

All the patients from 17 years old attending the PDCC with severe and/or spontaneous pain or deep caries in a permanent premolar or molar tooth were selected for possible enrollment (Figure 1). The exclusion criteria were medically compromised or pregnant patients. All the candidates were scheduled once a week for a check by the previously trained GPD, who completed a thorough clinical history and oral and systemic examination. A preoperative pulp and periapical diagnosis was established after clinical examination, thermal testing (Endo-Ice, Hygenic, Akron, OH, USA), and at least one periapical radiograph (Insight, Kodak, Rochester, NY, USA). 

**Figure 2:**
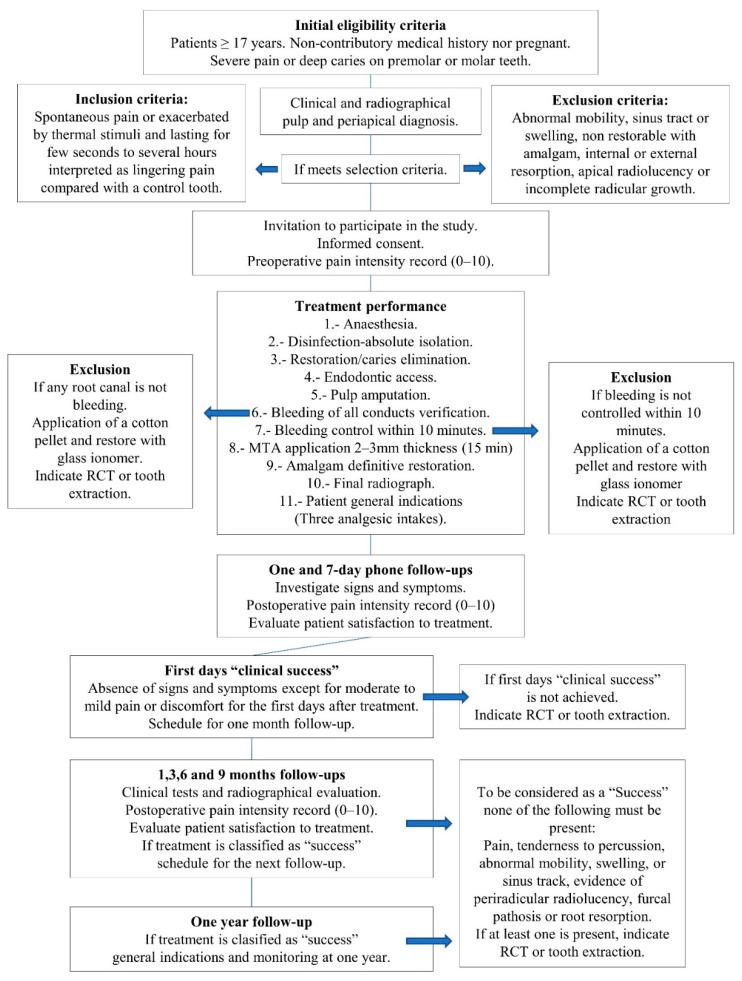
Workflow used by the general dentist at the public dental care clinic.

A numeric rating questionnaire was also used to record pain intensity (0 = no pain to 10 = worst pain imaginable). To invite the patient to participate, they should fulfill the following inclusion criteria: Preoperative symptoms of irreversible pulpitis, such as spontaneous pain or pain exacerbated by thermal stimuli, lasting for a few seconds to several hours, interpreted as lingering pain compared with a control tooth [34], and which could be reproduced using thermal testing. Teeth exclusion criteria were: pathological mobility, sinus tract, teeth that cannot be restored with amalgam, radiographic internal or external resorption as well as teeth with apical rarefaction or incomplete radicular growth. Forty-one volunteer patients were selected, informed in detail about the treatment, and written informed consent was obtained in accordance with the ethical principles of the Declaration of Helsinki (2013 version). Afterward, the patients were informed about the clinical procedures and potential risks. All their questions were solved, and it was clarified that this was not the conventional treatment for their diagnosis but an alternative. It was explained that continuous monitoring would be carried on, and they would need an RCT or a tooth extraction in case of failure. At all moments, the GPD followed the workflow diagram (Figure 2) supervised by an experienced endodontist who did not intervene.

### 2.3. Clinical Procedures 

All patients were treated in one visit by the same previously trained GPD. Firstly, local anesthesia was injected with 4% articaine with 1:100.000 epinephrine (Septanest, Septodont, Saint-Maur-des-Fossés, France). After applying the rubber dam (Nic tone, MDC Dental, Guadalajara, Mexico), disinfection of the tooth was performed using 2.5% sodium hypochlorite (NaOCl). Restauration, caries, and weak tissues were removed, and the endodontic access was prepared with a high-speed N° 4 carbide bur (Brasseler, Savannah, GA, USA,). When confirmed that pulp tissue was supplied with blood, most of it was removed with a sterile high-speed diamond bur (Brasseler, Savannah, GA, USA) under sterile saline solution irrigation and amputated to the canal orifices level using an endo excavator (33 L, Hu-Friedy, Chicago, IL, USA). If there was no bleeding, RCT was indicated and the patient was excluded. If bleeding occurred, hemostasis was achieved by rinsing the cavity with saline solution and applying small cotton pellets soaked in 2.5% NaOCl for a maximum of 10 min. White MTA (Angelus, Soluçöes Odontológicas, Londrina, Brazil) was mixed according to the manufacturer´s instructions and placed against the wound after hemostasis using an amalgam carrier (Pulpdent, Watertown, MA, USA) and packed using an amalgam standard condenser (American Dental, Missoula, MT, USA). The material was adapted to be 2 to 3 mm thick, and a moistened cotton pellet was placed directly over the MTA. After 15 min, the cotton was removed, and the tooth was restored with amalgam. A postoperative periapical radiograph (Insight, Kodak, Rochester, NY, USA) was taken after restoration with a radiographic holder Rinn XCP (Dentsply, Elgin, IL, USA) to maximize the reproducibility of the radiographic paralleling technique. General care instructions were given to the patients, and three analgesic intakes (non-steroidal anti-inflammatory drugs) were recommended. The endodontist supervised all the procedures, registering the time needed to perform them without his intervention. In addition, the patients were asked about their satisfaction with the treatment duration. The possible answers were: “Less than I expected,” “Just what I expected,” “More than I expected,” and “Much more than I expected.” Twenty-four hours and seven days after the procedure, patients were contacted by phone to record pain intensity, tenderness to mastication, need for intake of analgesics (more than the recommended), and satisfaction with the effect of the treatment. The possible answers for their satisfaction level were: “Very satisfied,” “Satisfied,” “Neither satisfied nor dissatisfied,” “Dissatisfied,” or “Very dissatisfied.” 

All the patients were scheduled for five follow-ups, and their satisfaction with the effect of the treatment was recorded. Clinical variables were persistent or spontaneous pain, tenderness to percussion or palpation, tooth discoloration, abnormal mobility, swelling, periodontal pocket, or sinus tract related to the treated tooth and evaluated at 1, 3, 6, 9, and 12 months. Radiographic variables were evidence of periapical radiolucency, furcal pathosis, or root resorption observed at the 3, 6, and 12 months radiographs, which were digitized by one member of the group (R.G.S.L.T) using a digital camera (Nikon, Tokyo, Japan) at a resolution of 1600 × 1200 pixels. Contrast and brightness were automatically adjusted using Adobe Photoshop 7.0 software (Adobe, San Jose, CA, USA), and digital black box images were positioned on the coronal third of the teeth so only the medium and apical thirds of each radiographic images were shown for evaluation to three previously calibrated observers who were blind to the aims of the study. All clinical and radiographic data were reviewed separately by two experienced endodontists (T.D. and R.A.D.P). A treatment was considered a success if none of the clinical or radiographic variables were present at the one year follow-up.

### 2.4. Statistical Analysis

The interexaminer consistency for radiographic variables was performed by the kappa coefficient (>0.81) using the statistical package IBM SPSS (Version 20.0 for Windows, NY, USA). Descriptive analyses were performed for quantitative variables including mean and standard deviations, while qualitative ones were expressed as frequencies and percentages. 

## 3. Results

Forty-one patients received the intervention in 35 molars (16 maxillary–19 mandibular) and six premolars (four maxillary—two mandibular) teeth. Their clinical characteristics are summarized in Table 1. The range of patients´ ages was 17 to 78, while females were the predominant gender. 

All the participants reported preoperative moderate or severe pain and tenderness to mastication. Twenty-four hours after the treatment, 87.8% of the patients reported no pain, and only one reported tenderness to mastication. On the seventh day, 97.5% reported no pain, and 100% reported the absence of tenderness to mastication. During the first days, 97.5% were completely satisfied with the treatment. Only one patient reported severe pain and tenderness to mastication on the third day. She was excluded and RCT was indicated. Therefore, forty patients were followed from this point.

**Figure 3:**
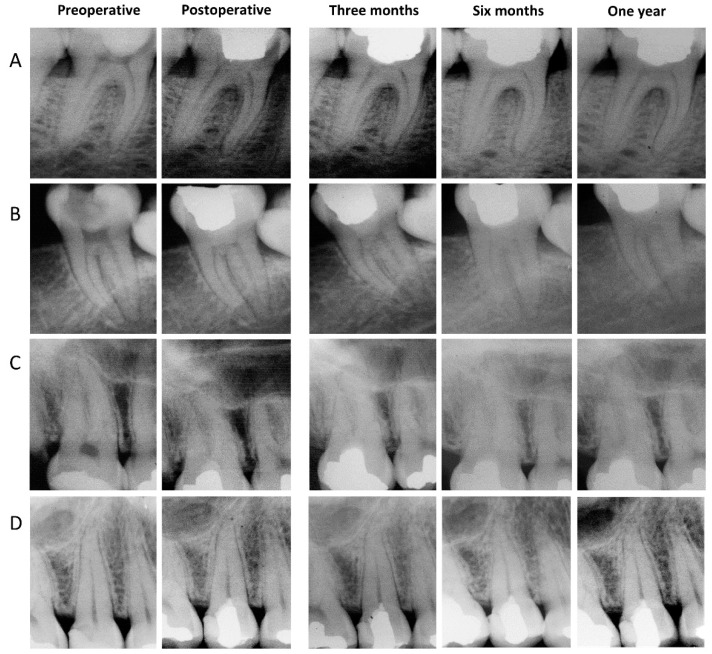
Preoperative, postoperative, and the three follow-up (3, 6, and 12 months) radiographs of four representative cases. None of them presented pain, tenderness to percussion or palpation, tooth discoloration, abnormal mobility, swelling, periodontal pocket, sinus tract, nor evidence of periapical radiolucency, furcal pathosis, or root resorption.

One patient reported slight tenderness to mastication at one-month follow-up, and an occlusal adjustment was performed. After three months and all subsequent follow-ups, 100% of the participants reported the absence of pain, and 100% reported being “Very satisfied” with the treatment. All cases were considered clinical and radiographic successes (Table 2 and Figure 3). Therefore, and taking into account the excluded participant, a high success rate and patient satisfaction were achieved (97.5%).

In addition, regarding the treatment performance, it was registered that the complete workflow could be easily followed and applied by the GPD and was completed within 45 to 60 min. In total, 80.5% of the patients considered the duration of the treatment “Less than I expected,” 14.7% “Just what I expected,” 4.8% “More than I expected,” and 0% “Much more than I expected.” 100% of the invited patients accepted participating in the study and assisted in their follow-ups. 

## 4. Discussion

Pain is considered the most common cause that prompts patients to seek dental care; this was evident in this study since 58.5% of the participants reported severe pain while the rest had moderate pain. In general, spontaneous or lingering provoked pain is accepted as indicative of irreversible pulp tissue changes [27]. However, it is an empirical guess based on clinical signs and symptoms because of the extent of pulp damage that has never been demonstrated; a poor correlation between clinical and histologic data has even been reported [35,36]. On the other hand, historically, a carious exposed pulp has been considered equivalent to irreversible pulp injury regardless of symptoms, and treatments such as RCT or tooth extraction have to be performed [34] as the only two choices to relieve pain.

There is no doubt about the relevance of RCT; in fact, we are living through the most rapid and extensive technological evolution in endodontics with some remarkable developments in technologies including endodontic imaging, root canal preparation, disinfection, and filling [37]. All these developments will influence the already high success rate of RCT, mainly when specialized endodontists perform the treatment. However, when a GPD performs the RCT without the support of technology, epidemiologic studies report that a high percentage of inadequately treated teeth are associated with apical periodontitis [38,39,40]. 

In Mexico and several other countries, an RCT performed by a specialist and assisted by current technology is considered a high-cost treatment that is not covered by public healthcare services [41]. The financial factor has been reported as the primary barrier to receiving an RCT [42], and socioeconomic status is a strong determinant of tooth loss [43]. Even in highly developed countries, a percentage of the population is more likely to experience tooth extraction and edentulism [44,45] due to financial issues. FP is economically more accessible than RCT, mainly because it is less invasive, technically more straightforward, requires less equipment and supplies, and can be performed in less time. It has already been recommended to implement it as a viable treatment in communities with economic limitations [41]. Moreover, it must be tried as an ethical treatment option when the patient is left with only the option of tooth extraction due to financial constraints [46]. 

Several studies indicated that FP might also lead to favorable outcomes in treating symptomatic teeth with irreversible pulpitis [3,47,48]. Furthermore, it could be a prospective substitute for RCT to treat permanent teeth with carious pulp exposures [33] or irreversible pulpitis where deeper inflammation is expected as it is considered a more predictable procedure due to the more extensive removal of inflamed tissue [20] compared to pulp capping or partial pulpotomy.

To consider the FP as an alternative to RCT, postoperative pain and the clinical and radiographic outcomes are fundamental. The treatment success in this study was assessed by combining three variables, patient satisfaction, clinical, and radiographic outcomes. A very high success rate (97.5% at 12 months considering the patient who was excluded three days after the procedure) was obtained in this study and it was consistent with the reports from previous studies evaluating the outcome of pulpotomy in mature permanent teeth [18,19,32,47,48]. The success of the treatments was primarily based on the absence of symptoms, with additional consideration of some signs such as abnormal mobility, sinus tract, furcal pathosis, and swelling. Due to the probed unreliable response to cold testing of pulpotomized teeth [49], the exact condition of the radicular pulp could not be clinically assessed. For this, the radiographic evaluation was a compliment to determine success, principally by the absence of furcal pathosis, root resorption, and periapical radiolucency as in other reports [23,33]. In addition, it has been reported that vital pulp therapy failures tend to occur during the first months [29]. While several studies have considered six-month follow-ups suitable for teeth treated with a pulpotomy [49,50,51], a 12-month follow-up period was adopted for a better evaluation of clinical and radiographic success rates and to concur with other investigations [52,53,54]. If the procedure had not been successful, follow-up examinations would have revealed the development of a periapical lesion or the recurrence of symptoms. Although a necrotic pulp can also be asymptomatic and radiographically inconspicuous, it has been postulated that a lack of clinical signs and symptoms, regular radiographic features, and physiologic function of the tooth after one year of follow-up can be considered successful [55].

Patient satisfaction regarding the effect of the treatment was assessed at each evaluation. At the first examination after 24 h, 90.3% of patients reported being “very satisfied”; the percentage increased to 97.5% after seven days. This was important and influenced the great acceptance from the patients who came to request attention at the PDCC, principally because of the opportunity to avoid tooth extraction. A 100% attendance rate was achieved at all follow-up visits and after one year, this lessened to a certain point what we recognize as the main limitation of this pilot study, the reduced number of participants. 

For a long time, it was suggested that FP is only adequate in young and immature permanent teeth because the rich blood supply of the pulp may contribute to high success rates [56] compared to old and more fibrous pulps. However, some studies have included patients up to 50 years old and reported the patient´s age without noting an impact on the outcome of the therapy [32,57,58,59]. Based on this evidence and because PDCC treats patients of all ages, it was decided to include patients from 17 years old with no age limitations. The present results are consistent with these authors. 

The high success rate achieved in this study, which is expected if FP is performed routinely, is also related to some factors. For example, cases with apical rarefaction that could influence and reduce the success of endodontic treatment [60] were excluded. Although there are some reports [18,61] of the resolution of preoperative periapical radiolucencies after FP, more evidence is needed at this stage before FP can be implemented in these cases.

Another critical factor was the intraoperative inclusion criteria of observing bleeding from all root orifices after eliminating coronal pulp and a maximum of 10 min until the bleeding had stopped. This proposal arose after observing that this is a variable topic, as studies have used hemostasis periods ranging from 2 to 25 min [16,18,48,62,63,64]. However, several variables may have an impact, such as the local anesthetics and vasoconstrictors and the protocol for hemostasis by compression with dried or wet cotton pellets using NaOCl at various concentrations, chlorhexidine, corticosteroids, or saline. A limit of 10 min to control bleeding was decided, mainly since it has been proposed that bleeding that cannot be stopped within one to 10 min indicates progression of the inflammation into the radicular pulp [20] or infected and necrotic pulp, contraindicating the procedure [30]. Moreover, more than 10 min would prolong the treatment, which should be performed quickly. The bleeding stopped within the first five minutes in most treatments. Regarding the hemostatic protocol, NaOCl was used because using other products (e.g., hemostatic agents or corticosteroids) could be misleading and “mask” the true inflammatory state of the pulp; also, they could increase the cost of treatment. Regarding the pulp capping material, most of the success reports of this non-traditional and conservative treatment emerge from the use of hydraulic calcium silicate-based types of cement, mainly MTA. In this first approach, it was decided to use MTA as the most evaluated pulpotomy material in permanent teeth with pulp exposures.

Concerning the quality of coronal restoration, it is another critical factor that influences the success of endodontic treatment [65]. It can be essential to the long-term maintenance of vitality and function of the pulpotomized tooth [66]. It has been demonstrated that the most critical cause of failure is bacterial recontamination during the healing process [67]. Therefore, bacterial recontamination through coronal microleakage should be avoided by producing a well-sealed coronal restoration. 

Amalgam or resin composite were the only restorative options because Mexican public healthcare services could not provide custom crowns or inlays, and patients could hardly pay for them in private practice. In addition, it is well known that many patients with temporary restoration do not permanently restore their teeth once the pain has disappeared. For that reason, it was decided to restore the teeth with amalgam in the same session, as it requires less technical skills from the operator and still has more longevity and a lesser number of secondary caries compared to resin composite restorations [68]; in this way, the best possible long-term sealing was ensured.

On the other hand, considering the limitations of this study; the fact of only including systemically healthy patients delimits the results to this population, and therefore it is necessary to investigate whether the excellent success rate of this proposal can also be present in patients with systemic diseases, such as diabetics, or the immunosuppressed. 

We also believe that the high success rate in this study was due in part to the fact that the specialist supervised all the procedures, and although he did not intervene directly, his presence could even influence the GPD to follow the workflow and respect the selection criteria strictly. This could be a limitation if FP is implemented as a routine treatment in the PDCC. 

From the perspective of the GPD, the plain identification of bleeding and its hemostasis in each root canal of a multicanal tooth was indicated as the only critical component during the performance of the treatment. In fact, after analyzing the case of the patient who was excluded days after the treatment, it was indicated that there was doubt regarding the bleeding of one canal, possibly because this radicular pulp was inflamed or even necrotic. Therefore, emphasis should be given to the importance of following the workflow and strictly adhering to the selection criteria during the training of the GPD. 

## 5. Conclusions

This first approach of feasibility study demonstrated an excellent success rate and patient satisfaction when a GDP performed the FP in a PDCC in Mexico. It could be suitable as an option for symptomatic irreversible pulpitis or caries pulp exposure. The multicentric feasibility study will indicate if it can be successfully replicated with the same results to definitely establish if it could be considered as a routine treatment in the PDCC. This proposal could be replicated by public healthcare services from other countries that have the same needs and limitations.

## Figures and Tables

**Table 1:** Clinical characteristics of the patients and included teeth.

Group	Included Participants (*n* = 41)
	Mean ± S.D. (Range)
Age (years)	34.63 ± 15.76 (17–78)
Gender	Frequency (%)
Female	32 (78.0)
Male	9 (22.0)
Type of teeth	Frequency (%)
Premolars	6 (14.6)
Molars	35 (85.4)

**Table 2:** Preoperative and postoperative evaluations.

	Preoperative (*n* = 41)	Postoperative			
		1 Day (*n* = 41)	7 Days (*n* = 41)	1 Month (*n* = 40)	3, 6, 9, and 12 Months (*n* = 40)
Pain intensity (Rating 0–10)	Frequency (%)				
None (0)	0	36 (87.8)	40 (97.5)	40 (100)	40 (100)
Mild (1–3)	0	4 (9.8)	0	0	0
Moderate (4–6)	17 (41.5)	1 (2.4)	0	0	0
Severe (7–10)	24 (58.5)	0	1(2.5) *	0	0
Satisfaction with the effect of the treatment	Frequency (%)				
Very satisfied	------	37 (90.3)	40 (97.5)	40 (100)	40 (100)
Satisfied	------	3 (7.3)	0	0	0
Neither satisfied nor dissatisfied	------	0	0	0	0
Dissatisfied	------	1 (2.4)	0	0	0
Very dissatisfied	------	0	1 (2.5) *	0	0
Tenderness to mastication	41 (100)	1 (2.4)	0	1 (2.5)	0
Intake of analgesics	34 (82.9)	------	2 (4.9)	0	0

* Excluded patient on the third day. She was not considered in subsequent follow-ups due to the impossibility of evaluating the variables. However, 40 of 41 (97.5%) cases were considered successful when considering this case in the final success rate.

## Data Availability

The dataset used and analyzed during this study is available from the corresponding author upon reasonable request.
